# Free-radical scavenging properties of low molecular weight peptide(s) isolated from S1 cultivar of mulberry leaves and their impact on *Bombyx mori* (L.) (Bombycidae)

**DOI:** 10.1186/2049-1891-5-16

**Published:** 2014-03-11

**Authors:** Suchisree Jha, Palash Mandal, Phalguni Bhattacharyya, Amitava Ghosh

**Affiliations:** 1 Department of Botany, University of North Bengal, Siliguri 734013, West Bengal, India; 2 Department of Botany, Malda College, University of Gour Banga, Malda 732101, West Bengal, India; 3 Department of Botany, Asutosh College, 92, S.P. Mukherjee Road, Kolkata 700 026, West Bengal, India

**Keywords:** Antioxidant, HPLC, Low molecular peptide(s), Mulberry leaf, Silkworm

## Abstract

The mulberry leaves have been considered as a sole food source for silkworm, *Bombyx mori* (L.). In present work an attempt was made to investigate the role of low molecular weight peptide(s) isolated from mulberry leaves on silkworm rearing. Also we have tried to find out the role of free-radical scavenging activities of isolated peptide(s) on silkworm growth. Larval growth rate was found effective under the influence of peptide(s). Consumption rate of larvae after peptide(s) treatment on mulberry leaves was significantly enhanced over control. High antioxidant activity was found in Low molecular weight peptide(s) which have an effect on silkworm.

## Background

*Bombyx mori* L. also commonly known as silkworm is a monophagous insect reared in captivity (sericulture). India is the second largest producer of silk and also the largest consumer of silk in the world followed by China [1]. Human beings have benefited by the silkworm in various ways and scientists have been continuously trying to improve the techniques of silkworm rearing. The mulberry leaves have considered as a sole source of food for silkworm, especially at larval stage. The quality and quantity of mulberry leaves determine the growth and development of silkworm and subsequently cocoon production [2]. Plants are considered as a richest resource of phytochemicals and these phytochemicals have been reported to manipulate the life cycle and activity of different insects [3,4]. The effects of different types of dietary protein on silkworm growth were resolute by using semi-synthetic diets. Some workers have clearly described that protein acts as an essential ingredients in silkworm diet for their growth and silk production. Several reports stated that the soybean meal as a protein source in silkworm diet can significantly increase the weight of silkworm larvae and fresh silk glands [5,6]. Since smaller proteins have also been considered as peptides, therefore it may be predicted that these peptides may also have significant effect on the growth and development of silkworm. In present study, a scientific attempt was made to figure out the effect of peptide(s) of two different molecular ranges (0.5-3 kDa and 3-10 kDa) isolated from mulberry (S1 cultivar) leaves of different maturation stages on silkworm growth and silk production.

## Material and methods

### Plant culture

Leaves of S1 cultivars of mulberry leaves were collected from sericulture farm of Malda, West Bengal, India at same season and same time. Leaves were selected at different maturity status e.g. young, mature and senescence leaves and was weighed out (1 kg each). Young mature and senescence leaves were selected on the basis of the biological (i.e. Chlorophyll content and protein content) and the morphological attributes of the leaves (i.e. length and breadth).

### Isolation and purification of low molecular weight peptide(s)

#### Preparation of extraction

One kg of leaves of each set were washed separately and thoroughly under tap water and cut into pieces. After repeated wash sets were treated with 0.2% Sodium hypochloride solution to avoid excessive contamination and finally washed with distilled water. The leaf pieces were separately crushed for peptides isolation in presence of Liq. N_2_ by a grinder and extracted with chilled distilled water with a measured amount by blender at 4˚C in cold room. To remove the unwanted materials, the extract was centrifuged at 10,000 rpm for 30 min using protease inhibitor PMSF at 4˚C. The supernatant was collected and stored in deep freeze (-20˚C).

#### Ether washes

The extracts were subjected to ether wash at acidic pH to remove endogenous hormonal impurities, fats, lipids and oil as impurities.

#### Ion exchange chromatography

The extracts were passed through separate cation exchanger resin (900 meq-Sigma Chemical Co. USA filled in glass column 60 cm × 2.9 cm, 1.6 meq/mL) to get anionic hormone free solution, like indole acetic acid (IAA), abscisic acid (ABA), gibberellic acid (GA_3_). Solution of each sample held up some basic compound like cytokinin, amino acids, peptide and related compounds after coming from cation exchanger resin column. The resin was taken out from column and was neutralized with ammonia with constant shaking to avoid exothermic reaction and again reloaded it in the column, further eluted with 3 (N) NH_4_OH in cold. The total liquid was dried in freeze with liquid N_2_ trap to remove ammonia. The sample obtained after cation resin processing was taken in water with little acidic pH and again passed through anion resin (700 meq-Sigma Chemical Co. USA filled in glass column 60 cm × 2.9 cm, 1.6 meq/mL). Again it washed, neutralised and eluted with 1 (N) HCl. After passing through this column, basic compounds and cytokinins were removed from solution. As a result, liquid samples were free from electrolytes, only amphoteric compounds, peptides and amino acids were present in each collected sample. Then concentrated aqueous acidic solutions were washed 4 times with equal volume of peroxide free ether to remove traces of IAA, ABA, and GA.

#### Ultra-filtration

After discarding of anionic hormones, the extracts were filtered through Millipore ultra filtration system with Amicon filters 10 kDa (YM 10), 3 kDa (YM3) and 0.5 kDa (YC 05) cut off with 1.5 kg/cm^2^ N_2_ gas pressure. The samples were repetitively filtered and finally purified and dry extracts were obtained which were semisolid and brownish in nature. The obtained peptide extract was dissolved in 50 mL distilled water and stored in freeze at -20°C for further analysis.

#### HPLC analysis

The semi purified concentrated peptide(s) from different maturity status of leaves were passed through C18 HPLC, Waters™ 486 reverse phase column in 10% Methanol as running solvent fitted with 515 HPLC pump, running time 60 min, absorbance at 250 nm, column length 3.9 mm × 150 mm, injection volume 20 μL, flow rate 0.5-1.0 mL/min, pump pressure 4000 psi., and purified. The peptide(s) appeared at different retention time were repeatedly tried and purified, concentrated and collected in deep freeze under -20°C. Each peak was isolated with their retention time and re-injected into the column to check its repetitive occurrence. Thus these peaks were concentrated and used to study their effect on silkworm.

### Feeding trail

#### Experimental insect and rearing method

The present experiment was conceded in the laboratory through the well established methods [7]. The diseases free laying (DFL) of the silkworm breed preferred for the experiment was a F1 hybrid (Nistari × bivoltine), collected from Sericulture Farm of Malda, West Bengal, India. The fifth instar larvae were utilized for the treatment. Healthy and fresh leaves of S1 cultivars of mulberry were used in the present research work. The leaves were collected from the nearest Sericulture Farm and stored cool to maintain its freshness.

Prior to the initiation of silkworm rearing the rearing room, plastic tray, and other materials used for rearing was carried out as preventive measure against pathogens. In a plastic tray, rearing of ten caterpillars was conducted by feeding with S1 mulberry cultivars as a control treatment. In parallel ways six different set were established for different peptide(s) treatment.

#### Leaf treatment

Peptide(s) isolated from young (P_y_), mature (P_m_), and senescence (P_s_) leaves in both range 0.5-3 kDa and 3-10 kDa was 20 times diluted by distilled water. Leaves were soaked in peptide(s) for 30 min before feeding them to the larvae and air-dried for 15 min and given to silkworm. Six separate groups, with 10 larvae were kept and feed by different peptide(s) treated leaves in separate plastic tray.

#### Rearing bed maintenance procedure

Total rearing was performed at a temperature of 28 ± 1°C and a relative humidity of 70 ± 5%. During feeding period, known quantity of peptide(s) treated leaves served in treatment set and only fresh S1 leaves served in control set four times per day. The trays were placed under adequate ventilation. Disinfection of the room was strictly maintained at rearing time. Hands were sterilized with dettol solution before handling the worms during the rearing time. To maintain room temperature and humidity one thermo-hygrometer was used near the larval bed. The grass of larvae was continuously discarded from the tray. Dead larvae if found, during the rearing period were immediately removed.

#### Data collection

The weight of larvae in each tray was monitored by weighing them on weighing balance daily and the growth rate pattern of caterpillar was calculated. When larvae started spinning they were left uninterrupted for four to five days to form the cocoon. After complete cocoon formation, the weight of cocoon of each set was measured. Cocoon shell weight also was measured after release of the moth from cocoon shell. Weight of male and female moth was recorded separately. Number of eggs laid by per pair of moth in each set was noted. Growth index, shell ratio, effective rearing rate (ERR) were calculated by formulae (given below). The collected data was subjected for graphical and statistical analysis [8].

$$\mathrm{Shell}\;\mathrm{ratio}\;\left(\%\right)\;=\;\frac{\mathrm{Single}\;\mathrm{shell}\;\mathrm{weigth}\;\left(\mathrm{gm}\right)}{\mathrm{Single}\;\mathrm{cocoon}\;\mathrm{weight}\;\left(\mathrm{gm}\right)}\;\times \;100$$

$$\mathrm{ERR}\;\%=\;\frac{\mathrm{Total}\;\mathrm{no}.\;\mathrm{of}\;\mathrm{cocoons}\;\mathrm{harvested}}{\mathrm{Total}\;\mathrm{no}.\;\mathrm{of}\;\mathrm{larvae}\;\mathrm{brushed}}\;\times \;100$$

$$\;\mathrm{Weight}\;\mathrm{of}\;\mathrm{single}\;\mathrm{cocoon}=\;\frac{\mathrm{Weight}\;\mathrm{of}\;5\;\mathrm{male}\;\mathrm{cocoons}+\mathrm{Weight}\;\mathrm{of}\;5\;\mathrm{female}\;\mathrm{cocoons}\;\left(\mathrm{gm}\right)}{\mathrm{No}.\;\mathrm{of}\;\mathrm{cocoons}\;\mathrm{taken}\;\left(10\right)}$$

$$\mathrm{Single}\;\mathrm{shell}\;\mathrm{weight}=\frac{\mathrm{Total}\;\mathrm{shell}\;\mathrm{weight}\;\mathrm{of}\;5\;\mathrm{male}\;\mathrm{cocoon}+5\;\mathrm{female}\;\mathrm{cocoon}\;\mathrm{shell}\;\left(\mathrm{gm}\right)}{\mathrm{Total}\;\mathrm{no}\;\mathrm{of}\;\mathrm{cocoons}\;\mathrm{taken}\;\left(10\right)}$$

### Determination of antioxidant activity of isolated peptide(s)

#### DPPH -Scavenging activity

DPPH is a relatively stable free radical which has been widely used to examine the free radical-scavenging activity of tested samples. The radical scavenging activity of the aqueous extracts was measured by DPPH method [9]. The reaction mixture contained 1.8 mL of 0.1 mmol/L DPPH and 0.2 mL of aqueous extracts. The reaction mixture vortexes and kept in the dark at room temperature for 30 min. The absorbance was measured at 517 nm. A reaction mixture without test sample was taken as control. The free radical scavenging activity of tested sample were expressed as percentage of inhibition and were calculated according to these equation:

$$\%\;\mathrm{inhibition}\;\mathrm{of}\;\mathrm{DPPH}\;\mathrm{activity}=\left[\left(A_{0}-A_{1}\right)/A_{0}\right]100\;\%$$

Where A_0_ is the absorbance value of the blank sample i.e. control reaction. And A_1_ is the absorbance value of the tested sample. A curve of inhibition percent or percent scavenging rate against samples concentrations was determined from where IC_50_ (concentration of the sample required to inhibit 50% of free radicals) of tested sample were calculated.

#### ABTS^+^ scavenging activity

The spectro-photometric analysis of ABTS^+^ radical cation(s) scavenging activity was determined according to Re *et al.*[10] method with some modification. This method is based on the ability of antioxidants to quench the ABTS radical cation, in a blue/green chromophore with characteristic absorption at 734 nm, in comparison to that of BHT (Butylated Hydroxy Toluene). The ABTS^+^ was obtained by reacting 7 mmol/L ABTS^+^ radical cation(s) in H_2_O with 2.45 mmol/L potassium persulfate (K_2_S_2_O_8_), stored in the dark at room temperature for 12-16 h. Before usage, the ABTS^+^ solution was diluted to get an absorbance of 0.750 ± 0.025 at 734 nm with sodium phosphate buffer (0.1 mol/L, pH 7.4). Then, 2 mL of ABTS^+^ solution was added to 1 mL of the aqueous extract. After 30 min, absorbance value was recorded at 734 nm, relative to a blank absorbance. The percentage inhibition of the samples was calculated as:

$$\mathrm{Inhibition}\;\%=\left[\left(A_{0}-A\right)/A_{0}\right]\times 100$$

Where *A*_0_ is the absorbance at 734 nm of the control, *A* is the absorbance at 734 nm of the sample mixture.

#### Reducing power

The assay was performed according to the method of Oyaizu [11] with some modification. Extracts were diluted at different concentration 2.5 mL of the 0.2 mol/L phosphate buffer (pH: 7.0) and 2.5 mL of 1% potassium ferricyanide solution were added with tested sample and vortexed. The mixtures were incubated at 50°C for 20 min in a water bath. The tubes were cooled at room temperature and 2.5 mL of 10% trichloroacetic acid was added and centrifuged at 3,000 rpm for 10 min. 2.5 mL upper layer was mixed with 2.5 mL of distilled water and 250 μL of 0.1% aqueous ferric chloride. Fluorescent green colour was appeared and absorbance of the final solution was recorded at 700 nm.

#### Nitric oxide scavenging assay

Nitric oxide was generated from sodium nitroprusside and measured by the Greiss reaction [12]. 320 μL extract, 360 μL (5 mmol/L) sodium nitroprusside-PBS solutions, 216 μL Greiss reagent (1% sulfanilamide, 2% H_3_PO_4_ and 0.1% napthylethylenediamine dihydrochloride) was mixed and incubated at 25°C for one hour. Finally 2 mL water was added and absorbance was taken at 546 nm.

Radical scavenging activity was expressed as percentage inhibition from the given formula:

$$\%\;\mathrm{inhibition}\;\mathrm{of}\;\mathrm{NO}\;\mathrm{radical}=\left[\left(A_{0}–A_{1}\right)/A_{0}\right]\times 100.$$

Where A_0_ is absorbance of control and A_1_ is the absorbance of sample.

#### Superoxide anion radical scavenging activity

The superoxide radical scavenging activity was measured by the method of Nishikimi *et al.,*[13] with slight modification. All solutions were prepared in 0.05 mol/L phosphate buffer (pH 7.8). The photo-induced reactions were performed using fluorescent lamps (20W). The reaction mixture contained 1 mL of NBT solution (312 μmol/L prepared in phosphate buffer, pH- 7.4), 1 mL of NADH solution (936 μmol/L prepared in phosphate buffer, pH-7.4), and 1 mL of methanolic extract of different concentration. After 5 min incubation, 100 μL of PMS (120 μmol/L prepared in phosphate buffer, pH-7.4) was added to the reaction mixture. The reactant was illuminated at 25°C for 30 min and the absorbance was measured at 560 nm against methanol as control. The inhibition percentage of superoxide anion generation was calculated by using the following formula:

$$\begin{array}{c} \mathrm{Superoxide}\;\mathrm{radical}\;\mathrm{scavenging}\;\mathrm{effect}\;\left(\%\right)\\ \;=\;[\mathrm{Abs}.\mathrm{of}\;\mathrm{control}\;–\;\mathrm{Abs}.\mathrm{of}\;\mathrm{sample}\\ /\mathrm{Abs}.\mathrm{of}\;\mathrm{control}]\;\times \;100. \end{array}$$

## Result

### Effect of peptide(s) on the silkworm rearing system

Essential nutrients in exact ratio are required to improve the growth and development of *B. mori*[14]. Sarker [15] noted significant improvement of silkworm larval growth upon feeding them with mulberry leaves supplemented with different nutrients. In our present study 5^th^ instar larval growth rate pattern was found to be improved under the influence of S1 peptide(s) isolated at different maturity stages of the leaves. Consumption rate of the larvae under peptide(s) treatment was increased significantly over control. Maximum larval growth rate was observed during 96 h in both range of peptide(s) treatment and as well as in control set.

**Figure 1:**
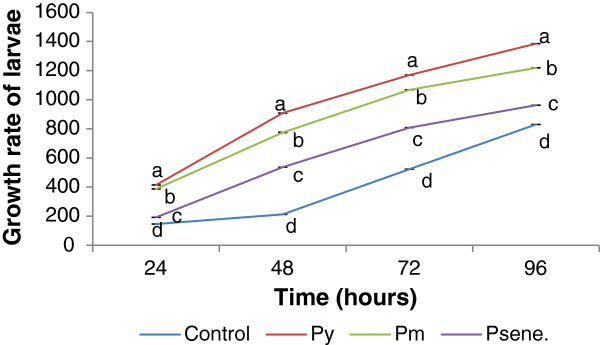
**Growth rate of larvae in S1 leaves treated with 0.5-3 kDa peptide isolated at different maturity stages of leaves (S1) and in control (only S1 leaves).** Results are represented as mean ± SEM, n = 3. Values with different letters (a, b, c & d) are significantly (*P* < 0.05) different from each other by Duncan’s multiple range test (DMRT).

**Figure 2:**
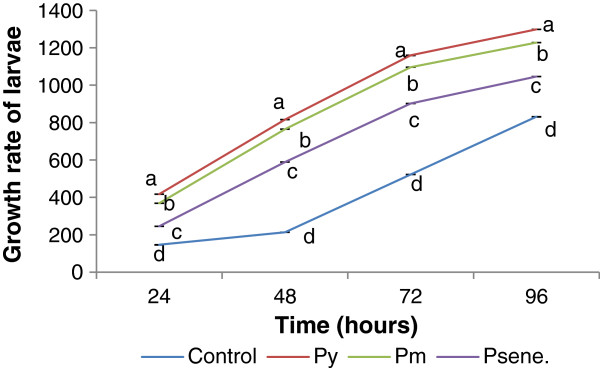
**Growth rate of larvae in S1 leaves treated with 3-10 kDa peptide isolated at different maturity status of leaves (S1) and in control (only S1 leaves).** Results are represented as mean ± SEM, n = 3. Values with different letters (a, b, c & d) are significantly (*P* < 0.05) different from each other by Duncan’s multiple range test (DMRT).

Highest larval growth rate was recorded at 96 h in P_y_ (0.5-3 kDa) followed by P_y_^3-10^, P_m_^0.5-3^, P_s_^0.5-3^, P_m_^3-10^, P_s_^3-10^ and control. From Figure 1 and Figure 2, it was noted that larval weight was gradually increased from 24 h to 96 h in each treatment set and control. So, it was evident that peptide(s) treatment elicits better growth potential for silkworm larvae. The 5^th^ instar silkworm larvae showed no significant difference in maintaining larval duration even in peptide(s) treatment.

Cocoon weight of the silkworm was highly improved by feeding the silkworm with mulberry peptide(s). Weight of single cocoon (g) was higher in P_y_^0.5-3^ followed by P_m_^0.5-3^, P_s._^0.5-3^, P_y_^3-10^, P_m_^3-10^, P_s._^3-10^, and control (Table 1). Maximum single shell weight (g) was recorded with P_y_^0.5-3^ in respect to control set. From the results (Table 1) it can be stated that a significant difference in the shell ratio (%) was occurred with P_y_ peptide(s) application followed by P_m_ and P_S_ in both molecular weight range over control.

**Table 1:** Effect of peptides of different molecular weight on various economical parameters of silkworm rearing system

**Items**	**Control (S1 leaves)**	**0.5-3 kDa**			**3-10 kDa**		
		**Py**	**Pm**	**Ps**	**Py**	**Pm**	**Ps**
Weight of single cocoon	0.66 ± 0 .027	0.79 ± 0 .017	0.78 ± 0.025	0.78 ± 0.020	0.77 ± 0.027	0.76 ± 0.027	0.7 ± 0.23
Weight of single Shell	0.1 ± 0.019	0.16 ± 0.024	0.13 ± 0.016	0.11 ± 0.018	0.15 ± 0.019	0.1 ± 0.022	0.1 ± 0.012
Shell ratio, %	15.15	19.75	16.26	14.65	19.61	13.65	14.29
ERR, %	90	100	100	90	100	100	80

Another commercial character ERR% (Effective Rearing Rate) was calculated from cocoon weight. Survivability was same in case of P_y_ and P_m_ treatment in both molecular weight ranges. 10% and 20% mortality rate was recorded by using P_s_^0.5-3^ and P_s_^3-10^ treatment respectively. Different seasonal effect showed no effective difference on mortality rate except a high death of larvae in rainy season (data not shown).

### Antioxidant activity of isolated peptide(s)

#### ABTS^+^ and DPPH scavenging activity

**Figure 3:**
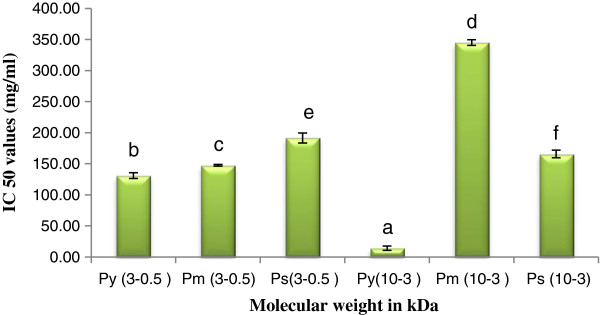
**ABTS**^**+ **^**scavenging activity of peptide(s) at different molecular weight.** Results are represented as mean ± SEM, n = 3. Values with different letters (a, b, c, d, e & f) are significantly (*P* < 0.05) different from each other by Duncan’s multiple range test (DMRT).

**Figure 4:**
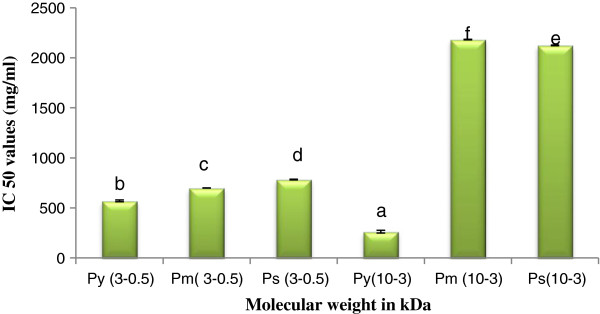
**DPPH scavenging activity of peptide(s) at different molecular weight.** Results are represented as mean ± SEM, n = 3. Values with different letters (a, b, c, d, e & f) are significantly (*P* < 0.05) different from each other by Duncan’s multiple range test (DMRT).

The ABTS^+^ and DPPH radical scavenging activity of peptide(s) of different molecular weight level are shown in Figures 3 and 4 respectively.

High scavenging activity was found in case of P_y_^0.5-3^ followed by P_m_^0.5-3^, P_s_^0.5-3^ in both ABTS^+^ and DPPH scavenging assay. Similarly in case of high molecular weight peptide(s), P_y_^3-10^ detected greater scavenging activity than P_m_^3-10^, and P_s_^3-10^. LMW peptide(s) exhibited high scavenging activity in both assays than HMW peptide(s). From the result it may be stated that antioxidant activity was augmented by the effect of LMW peptide(s).

#### Nitric oxide scavenging assay

**Figure 5:**
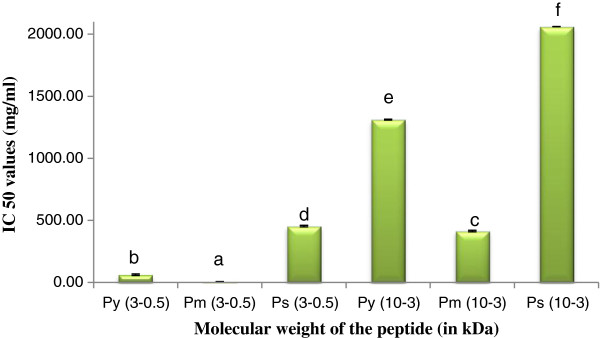
**Nitric-oxide scavenging activity of peptide(s) at different molecular weight.** Results are represented as mean ± SEM, n = 3. Values with different letters (a, b, c, d, e & f) are significantly (*P* < 0.05) different from each other by Duncan’s multiple range test (DMRT).

Nitric oxide is responsible for numerous physiological processes like neural signal transmission, vasodilation immune response etc. [16]. Our experiments revealed that P_m_ had higher scavenging activity rather than P_y_ and P_s._ The IC_50_ values of P_m_^0.5-3^ and P_m_^3-10^ were 5.11 mg/mL and 414.00 mg/mL respectively. IC_50_ values of other peptide(s) were shown in Figure 5.

Nitric oxide scavenging activities of peptide(s) were acted according to molecular weight dependent manner.

### Super oxide scavenging activity

**Figure 6:**
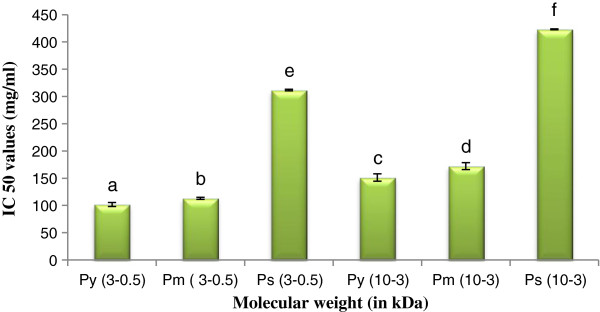
**Super oxide scavenging activity of peptide(s) at different molecular weight.** Results are represented as mean ± SEM, n = 3. Values with different letters (a, b, c, d, e & f) are significantly (*P* < 0.05) different from each other by Duncan’s multiple range test (DMRT).

Through a number of biological reactions superoxide radicals are generated. Superoxide radical anions act as potential precursors for highly reactive species like hydrogen peroxide and hydroxyl radicals. But they cannot initiate lipid oxidation directly [17]. Figure 6 showed the superoxide radical scavenging activity of the isolated peptide(s). P_y_ exhibited higher scavenging activity than P_m_ and P_s_ LMW peptide(s) had higher antioxidant activity than HMW.

### Reducing power activity

**Figure 7:**
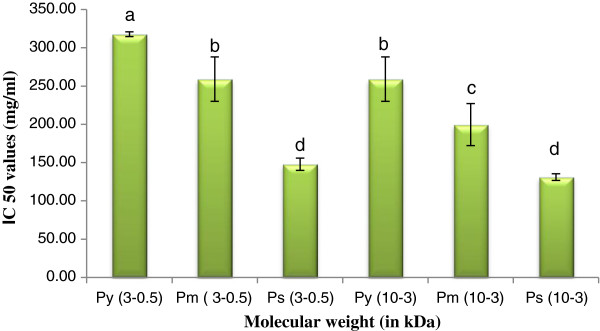
**Reducing power activities of peptide(s) at different molecular weight.** Results are represented as mean ± SEM, n = 3. Values with different letters (a, b, c & d) are significantly (*P* < 0.05) different from each other by Duncan’s multiple range test (DMRT).

To determine potential antioxidant activity of a biological compound, reducing power assay was performed where the reducing capacity plays as a significant indicator [18]. The ability of reducing power in each peptide(s) was determined with ascorbic acid equivalent. Higher ascorbic acid equivalent value indicates higher reducing ability of tested samples. P_y_ showed better activity than P_m_ and P_s_ (Figure 7). The LMW peptide(s) had significantly higher reducing power in comparison to those of HMW peptide(s). The results signify that smaller size peptide(s) exhibited better reducing power than high molecular weight fractions.

### Correlation of the antioxidant with silk rearing system

Considering our result, we have tried to correlate antioxidant activity of LMW peptide(s) with its effect on different growth and economically important developmental parameters of silkworm. From correlation matrix (Table 2) it can be concluded that a negative correlation was found in case of IC_50_ values of different scavengers like DPPH, ABTS^+^, Nitric oxide, and Super oxide with larval growth. Lowering of IC_50_ values indicate high antioxidant activity, which might be related with the growth and economic parameters of silkworm, as revealed from significant negative correlation. Only reducing power activity of peptide(s) have a positive correlation with larval growth rate, ERR%, weight of single cocoon shell and with shell ratio.

**Table 2:** showed correlation in between effect of peptide(s) antioxidant over different parameters of silkworm rearing system

**Items**	**Growth rate**	**ERR %**	**Weight of single cocoon, g**	**Weight of single shell, g**	**Shell ratio, %**
DPPH	−0.366	−0.494	−0.737	−0.830^*^	−0.798
ABTS	−0.271	−0.057	−0.138	−0.719	−0.786
Nitric oxide	−0.358	−0.717	−0.864^*^	−0.322	−0.158
Superoxide	−0.847^*^	−0.975^**^	−0.794	−0.704	−0.613
Reducing Power	0.927^**^	0.817^*^	0.679	0.899^*^	0.835^*^

^*^Correlation is significant at the 0.05 level (2-tailed).

^**^Correlation is significant at the 0.01 level (2-tailed).

### HPLC and peptide(s) sequencing

**Figure 8:**
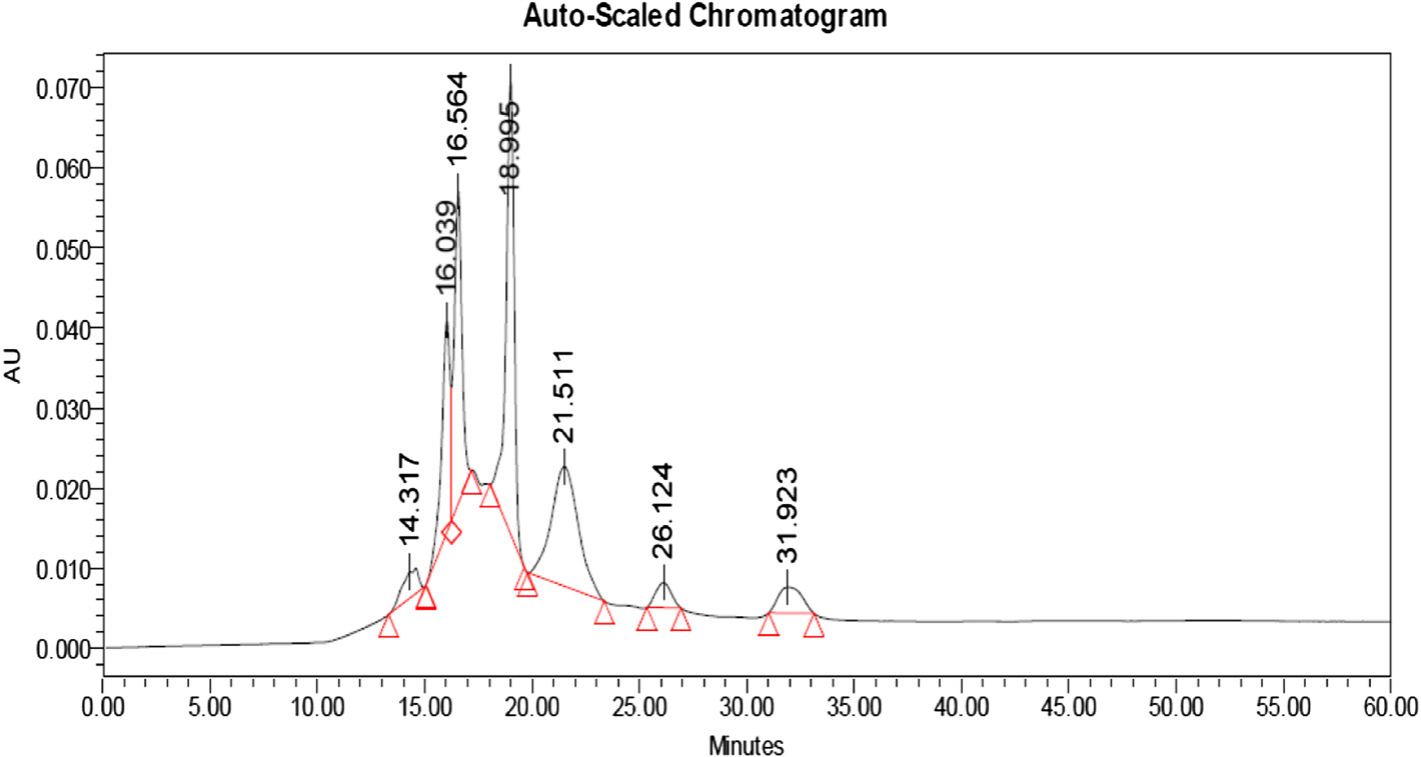
HPLC generated auto-scaled chromatogram of peptide(s) isolated from S1 young leaves.

**Figure 9:**
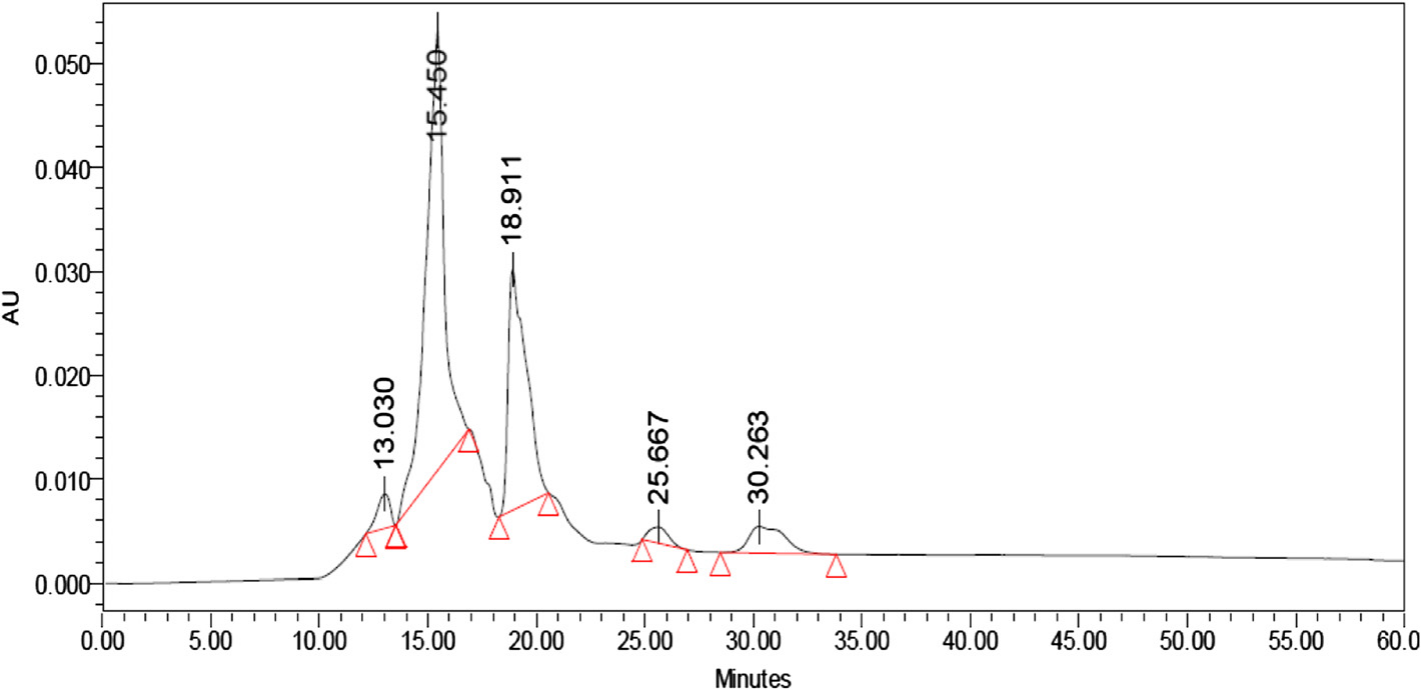
HPLC generated auto-scaled chromatogram of peptide(s) isolated from S1 mature leaves.

**Figure 10:**
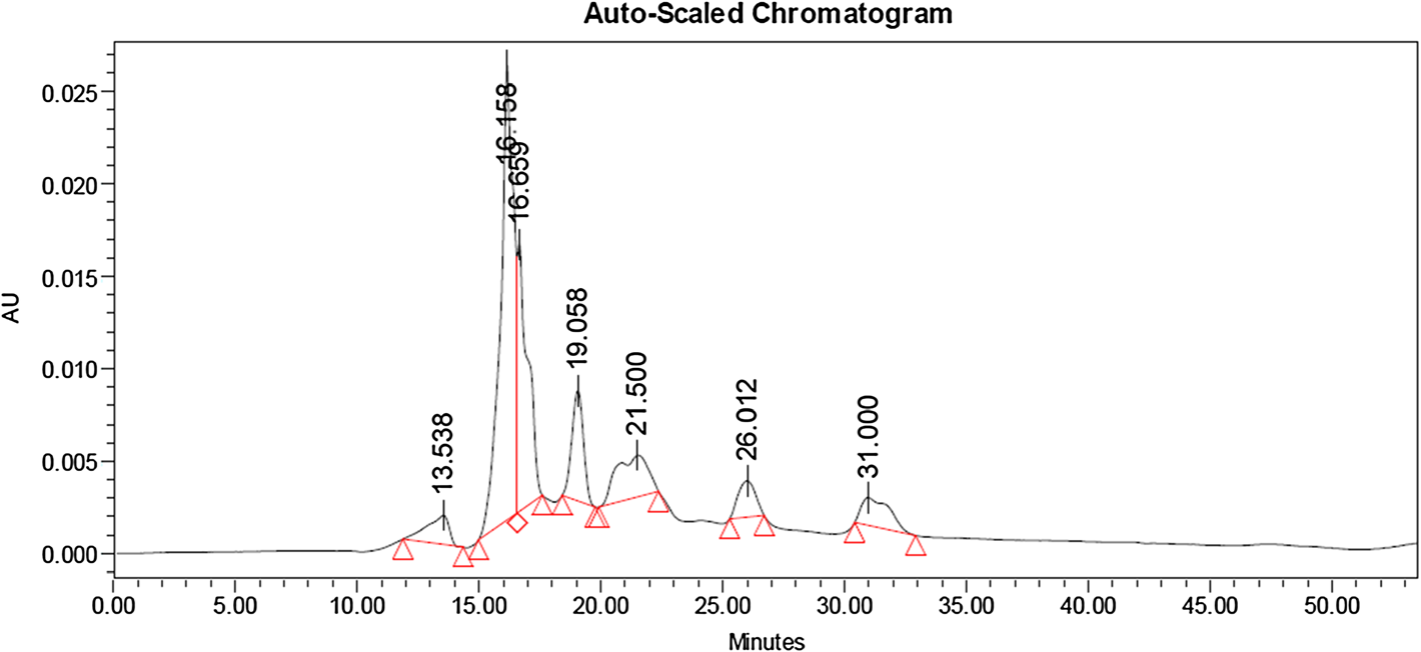
HPLC generated auto-scaled chromatogram of peptide(s) isolated from S1 senescence leaves.

In case of P_y_ and P_s_ seven peaks were shown but in P_m_ number of appeared peaks is 5 in HPLC chromatogram. The P_y_ appeared with one peak at 18.995 min in HPLC generated auto-scaled chromatogram whereas in case of P_m_ it was at 18.911 min, but such kind of single peak with highest area was not recorded in P_s_ (Figures 8, 9 and 10).

## Discussion

### Effect of peptide(s) on the biological parameters of the silkworm

In our observation larval weight was regularly increased from 24 h to 96 h in each peptide(s) treatment set. Similar result was obtained with folic acid administration where larval growth was influenced by folic acid (from 24 h onwards) but folic acid inserted no difference in larval duration of the 5^th^ instar silkworm [19]. Increment in larval weight was observed by Nirwani and Kaliwal [20], when silkworm was fed with different vitamins treated mulberry leaves. Cocoon weight is considered as an important commercial character because it is used to determine approximate amount of the raw silk but shell weight cannot be used as commercial feature because it damages the cocoon [21]. From the difference between the cocoon and cocoon shell weight, we get the weight of the pupa [22]. Subburathinan *et al.,*[23] was observed the fortification of mulberry leaves with calcium chloride to increase the cocoon characters like cocoon weight, shell weight cocoon, shell ratio and silk proteins. Jeyapaul *et al.,*[24] were recognized the highest shell weight when larvae were fed with *Coffea arabica* leaf extract fortified mulberry leaves. Different concentration of Tapioca flours and wheat suspension along with mulberry leaves showed superior shell weight [25]. Protein supplemented mulberry leaf have significant effect on larval growth and different economical parameter of silkworm [26]. Different nutrient formulation affects on the cost benefit ratio in sericulture directly or indirectly. In the present work, peptide(s) isolated from mulberry leaves have a beneficial effect on the silkworm rearing system. LMW peptide(s) supplemented with mulberry leaf have a significant role on larval growth, cocoon weight, and ultimately silk production.

### Antioxidant activity

In our recent work LMW peptide(s) showed better response in DPPH and ABTS^+^ scavenging activity and superoxide scavenge in a molecular weight dependent manner. This result is supported by the previous works by Girgih *et al.,*[27] in hemp seed and Aluko and Monu, [28] in quinoa seed, in which LMW peptide(s) fractions had higher DPPH scavenging activities than HMW peptide(s). Li *et al*., [29] explained that LMW peptide(s) from chickpea protein hydrolysates exhibited strong superoxide radical scavenging activity. In our study reducing power activity of LMW peptide(s) also appeared better. In contrast, Girgih *et al*., [27] reported that the reducing power activity was improved with the increase in molecular weight of peptide(s) in hemp seed protein. Cellular antioxidants can influence growth and development of plant by modulating mitosis processes and cell elongation to senescence and also death [30-32]. Antioxidant has a role in larval growth and subsequent silk production. From our result we can conclude that antioxidant activity of different scavengers (DPPH, ABTS^+^, Nitric oxide etc.) is inversely proportional with their IC_50_ values. We are observing from Table 2, that a negative correlation occurs in between IC_50_ values of peptide(s) in scavenging assay with growth and economical parameters of silkworm. Only reducing power showed positive result. It is natural, because it is ascorbic acid equivalent. As reducing power antioxidant can help in decreasing free ions present in the larval body, larvae can spin more silk and those the weight of single cocoon shell increased with reducing power activity. Other possibility may be that the reducing power directly or indirectly affects on protein synthesis. In the larval body it may increase the silk protein synthesis and subsequently increase shell weight. Shell weight depends on amount of raw silk. On the other hand it also can be stated that free radical in larval body may be inhibited their growth and silk production. When larvae fed antioxidant reached peptide(s) treated leaves, their silk production automatically enriched. From our result we can be acknowledged that if farmers used more antioxidant enriched substrate as a food for silkworm rearing, the production of silk will subsequently increased. However, more investigation will be required on this for clarification of the above statement.

### HPLC

The HPLC chromatogram profile of derived peptide(s) extract from different maturity status of mulberry leaf clearly exhibited the different segmental appearance according to desired molecular weight separation. However, this separation based on the retention time also helped us to purify the exact peptide(s) fractions and concentrated it for repeated cycles.

It is our observation that LMW peptide(s) may be a good supplement in feeding the silkworm along with the Mulberry leaf and its effect may have some positive response on the quality of the silk. Peptide(s) isolated from S1 mulberry cultivars increased the feeding rate of larvae. Although, these peptides have some beneficial effect on silkworm growth and development, it is difficult to estimate the antioxidant modulation by those peptide(s) fractions.

## Abbreviations

LMW: Low molecular weight; HMW: High molecular weight; Py0.5-3, Pm0.5-3 and Ps0.5-3: Peptide isolated from young, mature and senescence leaves in between 0.5-3 kDa range respectively; Py3-10, Pm3-10 and Ps3-10: Peptide isolated from young, mature and senescence leaves in between 3-10 kDa range respectively; DFL: Diseases free laying; IAA: Indole acetic acid; ABA: Abscisic acid; GA3: Gibberellic acid; ERR: Effective rearing rate; DPPH: 1,1-diphenyl-2-picrylhydrazyl; ABTS+: 2,2-azino-bis-(3-ethylbenzothiazoline-6-sulfonic acid) diammonium salt); NBT: Nitroblue tetrazolium chloride; BHT: Butylated hydroxy toluene.

## Competing interests

None of the authors have any competing interest to declare.

## Authors’ contributions

All authors have made significant charity to design the research, all laboratory worked, analysis of data, and in drafting the paper. All authors read and agreed the final manuscript.
